# Uraemic Cardiomyopathy in Different Mouse Models

**DOI:** 10.3389/fmed.2021.690517

**Published:** 2021-07-14

**Authors:** Cheng Chen, Caidie Xie, Hanzhang Wu, Lin Wu, Jingfeng Zhu, Huijuan Mao, Changying Xing

**Affiliations:** ^1^Department of Nephrology, Jiangsu Province Hospital, The First Affiliated Hospital of Nanjing Medical University, Nanjing, China; ^2^Department of Medical Science, Yangzhou Polytechnic College, Yangzhou, China

**Keywords:** uraemic cardiomyopathy, mouse models, surgical method, adenine, GDF-15, FGF-23

## Abstract

Uraemic cardiomyopathy (UCM) is one of the most common complications in chronic kidney disease (CKD). Our aim was to compare characteristics of various UCM mouse models. Mice were assigned to the following groups: the pole ligation group, 5/6 nephrectomy group (5/6Nx), uninephrectomy plus contralateral ischemia followed by reperfusion group (IR), adenine group, and sham group. Mice were sacrificed at 4, 8, and 16 weeks after surgery in the pole ligation, 5/6Nx, and IR groups, respectively. In the adenine group, mice were sacrificed at 16 weeks after the adenine diet. The structure and function of the heart and the expression of fibroblast growth factor 23 (FGF-23) and growth differentiation factor 15 (GDF-15) in hearts were assessed. The mortality in the 5/6 Nx group was significantly higher than that in the pole ligation, IR, and adenine groups. Echocardiogram and histological examination showed cardiac hypertrophy in the adenine,5/6Nx, ligation group, and IR group. In addition, cardiac fibrosis occurred in all CKD modeling groups. Interestingly, cardiac fibrosis was more serious in the IR and adenine groups. FGF-23 expression in sham mice was similar to that in modeling groups; however, the GDF-15 level was decreased in modeling groups. Our results suggest that the four models of UCM show different phenotypical features, molding time and mortality. GDF-15 expression in the hearts of UCM mice was downregulated compared with sham group mice.

## Introduction

Cardiovascular disease is the most common complication of chronic kidney disease (CKD) and the main cause of death among dialysis patients (1–3). A large number of patients with kidney disease die within 3 years after the diagnosis of uraemic cardiomyopathy (UCM) (4). Among the many cardiovascular complications, UCM is characterized by left ventricular hypertrophy (LVH) and diastolic dysfunction (5). To better understand its pathogenesis and explore prevention and treatment methods, it is essential to establish an effective UCM animal model.

Although the rat is an extremely useful model for studying cardiomyopathy of renal failure (6, 7), there are currently many genetic manipulations in mice, and it is easier to perform additional genetic manipulations in the mouse system. The 5/6 nephrectomy (5/6Nx) (8–10), uninephrectomy plus contralateral ischemia followed by reperfusion (IR) (11), pole ligation (12) and adenine diet models (13) of uraemic cardiomyopathy in mice have been reported in recent years. However, mice are small, and surgery is more difficult than surgery in rats. At present, there is no research comparing the advantages and disadvantages of various mouse UCM models. Therefore, we performed this study to test the survival rate, convenience of the surgery, and degree and characteristics of heart damage in different mouse models.

## Methods

### Experimental Mouse Models of Uraemic Cardiomyopathy

**Figure 1:**
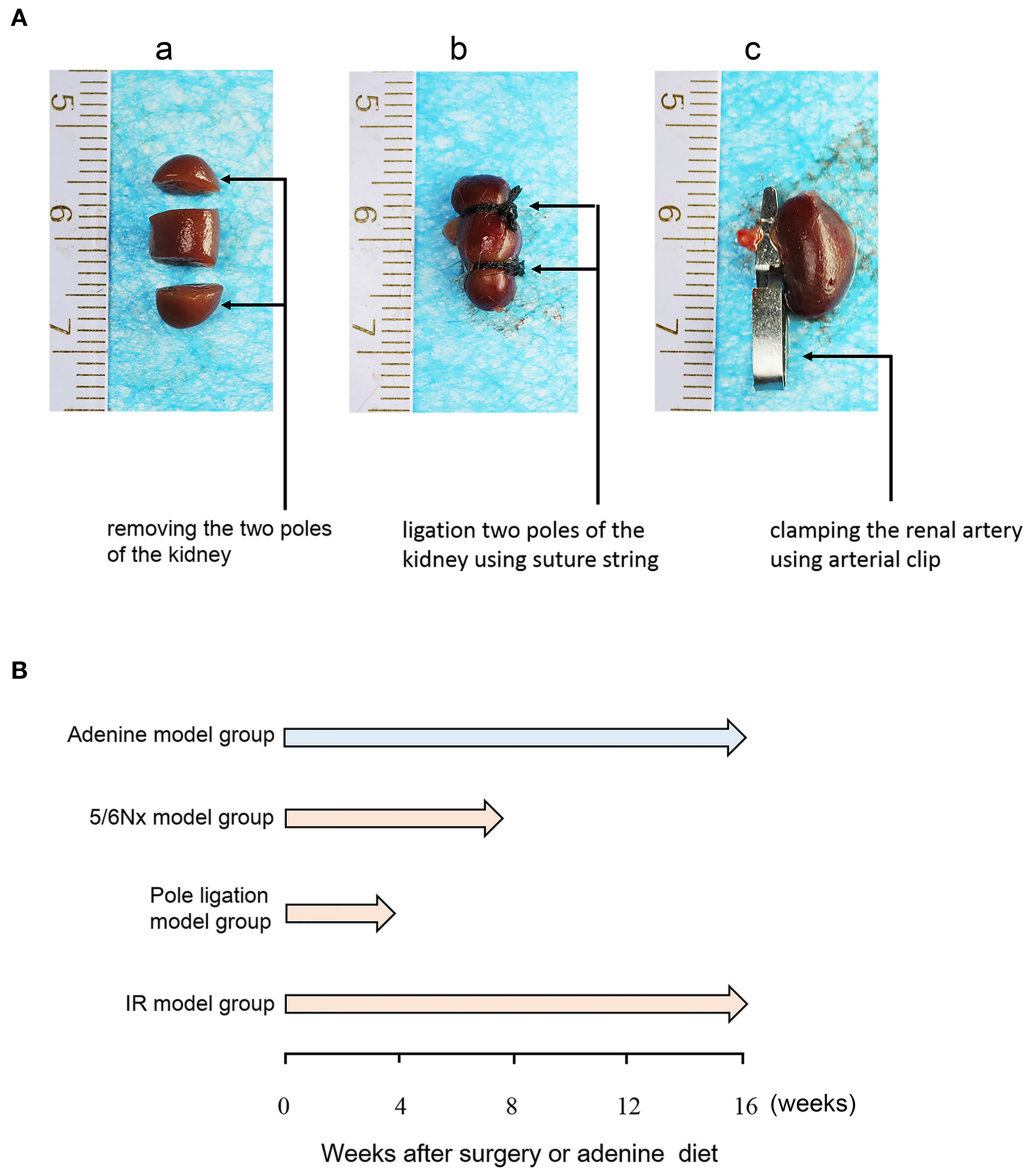
Protocols for the induction of UCM. **(A)** Diagrammatic drawing of surgery of the 5/6 nephrectomy group (5/6Nx), pole ligation group, and uninephrectomy plus contralateral ischemia followed by reperfusion group (IR). **(B)** Eight-week-old C57BL/6J male mice were randomly allocated to the sham (*n* = 14), 5/6th nephrectomy group (5/6Nx) (*n* = 45), pole ligation group (*n* = 34), uninephrectomy plus contralateral ischemia followed by reperfusion group (IR) (*n* = 20), and adenine group (*n* = 15). Mice were killed at 4, 8, and 16 weeks after surgery in the pole ligation, 5/6th nephrectomy, and uninephrectomy plus contralateral ischemia followed by reperfusion (IR) groups, respectively. In the adenine group, mice were killed at 16 weeks after the adenine diet (0.20%).

All animal surgical protocols and procedures were approved by the Animal Ethical and Welfare Committee of Nanjing Medical University. Male C57BL/6J mice were purchased from the Experimental Animal Center of Nanjing Medical University. All animals were maintained at 19–21°C on a 12-h/12-h light-dark cycle, fed a standard rodent diet and allowed free access to drinking water. Eight-week-old mice were randomly assigned to the following experimental groups: sham group, 5/6 nephrectomy group (5/6Nx) (Figure 1A,a), pole ligation group (Figure 1A,b), uninephrectomy plus contralateral ischemia followed by reperfusion group (IR) (Figure 1A,c), and adenine group. Under anesthesia (pentobarbital), 5/6Nx mice (*n* = 45) were produced by removing the two poles of the left kidney; haemostasis was achieved by electric cautery and pressure, and the right kidney was removed 1 week later. In the pole ligation group mice (*n* = 34), the two poles of the left kidney were ligated using a suture string and with appropriate force. After 1 week of recovery, the right kidney was removed. In the IR group (*n* = 20), the left kidney was subjected to 30-min of ischaemia followed by reperfusion on a warming platform. Subsequently, the right kidney was removed. The addition of adenine to the diet is a chemical method to induce CKD in mice. To investigate the effect of adenine on cardiac damage induced by CKD, mice (*n* = 15) were fed standard pellet chow containing 0.20% adenine (Sigma-Aldrich, St. Louis, MO) for 16 weeks. For sham animals (*n* = 14), a sham operation was performed whereby both kidneys were decapsulated and replaced intact. Mice in the pole ligation group and 5/6Nx group underwent non-invasive transthoracic echocardiography tests at 4 and 8 weeks after surgery, respectively, and the IR, adenine, and sham groups underwent tests at 16 weeks after surgery or adenine treatment. Then, mice were anesthetized with 50 mg/kg pentobarbital for sacrifice, with exsanguination performed by removing the eyeball. The heart and kidney were harvested from each animal and dissected for tissue analysis (Figure 1B).

### Serum Biochemistry

Blood urea nitrogen (BUN) and serum creatinine (Cr) levels were measured using a Hitachi 7180 auto analyser (Hitachi High Technologies Co., Tokyo, Japan). BUN and Cr concentrations were evaluated using urease-glutamate dehydrogenase and enzymatic methods, respectively.

### Haematoxylin-Eosin and Masson Staining

The hearts and kidneys of mice were perfused with 4% paraformaldehyde and fixed in 10% paraformaldehyde for 2 days before being sectioned. After staining heart tissue with haematoxylin-eosin (HE) and Masson and kidney tissue with HE, histological characteristics were observed under a light microscope.

### Echocardiography and Doppler Analysis

Transthoracic echocardiography was performed non-invasively with a high-resolution ultrasound imaging system (Vevo 2100; VisualSonics, Inc, Canada) equipped with a 30-MHz mechanical transducer. The mice were lightly anesthetized with 2% isoflurane/100% oxygen and placed on a warming platform (set to 37°C) for the duration of the recordings. Two-dimensional guided M-mode echocardiography was performed in the parasternal long-axis view. Left ventricular ejection fraction (LVEF), fractional shortening (FS), the ratio of left ventricular transmitral early peak flow velocity to left ventricular transmitral late peak flow velocity (the E/A ratio), left ventricular end-diastolic anterior wall depth (LVAWd), left ventricular end-systolic anterior wall depth (LVAWs), left ventricular end-diastolic posterior wall depth (LVPWd), left ventricular end-systolic posterior wall depth (LVPWs), and relative wall thickness (RWT) were measured. M-mode and Doppler measurement data represent 3–6 averaged cardiac cycles from at least two scans per mouse.

### Fibroblast Growth Factor 23 (FGF-23) and Growth Differentiation Factor 15 (GDF-15) Immunohistochemistry

Immunohistochemistry was performed on the hearts to analyse the protein expression of FGF-23 and GDF-15. Hearts were fixed in 10% paraformaldehyde and embedded in 80% methyl methacrylate (MMA) (BDH Chemicals, Poole, England) with 20% dibutylphthalate (Merck, Darmstadt, Germany) after dehydration in an increasing alcohol series. Three-micrometer-thick tissue sections were cut using a Polycut 2500 S microtome (Reichert-Jung, Nussloch, Germany). To remove MMA, tissue sections were incubated in 50% xylene and 50% chloroform. Subsequently, sections were rehydrated in a series of decreasing alcohol concentrations. Next, tissue sections were incubated with Tris-EDTA for 20 min and washed again three times with PBS buffer. Tissue sections were incubated with 5% bovine serum albumin to prevent non-specific binding of the secondary antibody. Subsequently, sections were incubated overnight at 4°C with polyclonal rabbit anti-GDF-15 (Proteintech, Wuhan, China, Catalog number: 27455-1-AP) and rabbit anti-FGF-23 (R&D, Minnesota, USA, Catalog numbers: 26291) at dilutions of 1:800 and 1:100, respectively. The next day, tissue sections were incubated with polyclonal 1:100 biotinylated goat-anti-rabbit (Zhongshanjinqiao, Beijing, China) for 1 h and with 1:200 horseradish peroxidase labeled streptavidin (Invitrogen, Life Technologies, Bleiswijk, The Netherlands) for 1 h. Chromogenesis was performed by treatment of the sections with AEC reagent (Invitrogen, Life Technologies, Bleiswijk, The Netherlands) for 6 min and by counterstaining with haematoxylin. Finally, the sections were mounted with ClearMount Mounting Solution (Invitrogen, Life Technologies, Bleiswijk, The Netherlands) and covered with a coverslip. Finally, microcosmic images were observed under a light microscope.

## Statistical Analyses

For continuous variables, data are expressed as the mean ± SEM. Statistical analysis was performed using unpaired, two-tailed *T*-test and one-way ANOVA with Tukey's multiple comparison test. For discrete variables, we used the chi-squared test. Statistical analysis was performed using SPSS 13.0 (SPSS, Inc., Chicago, IL, USA). A value of *P* < 0.05 was considered significant.

## Results

### Comparisons of Survival Rate, Pathology, and Kidney Function in Different Models

**Figure 2:**
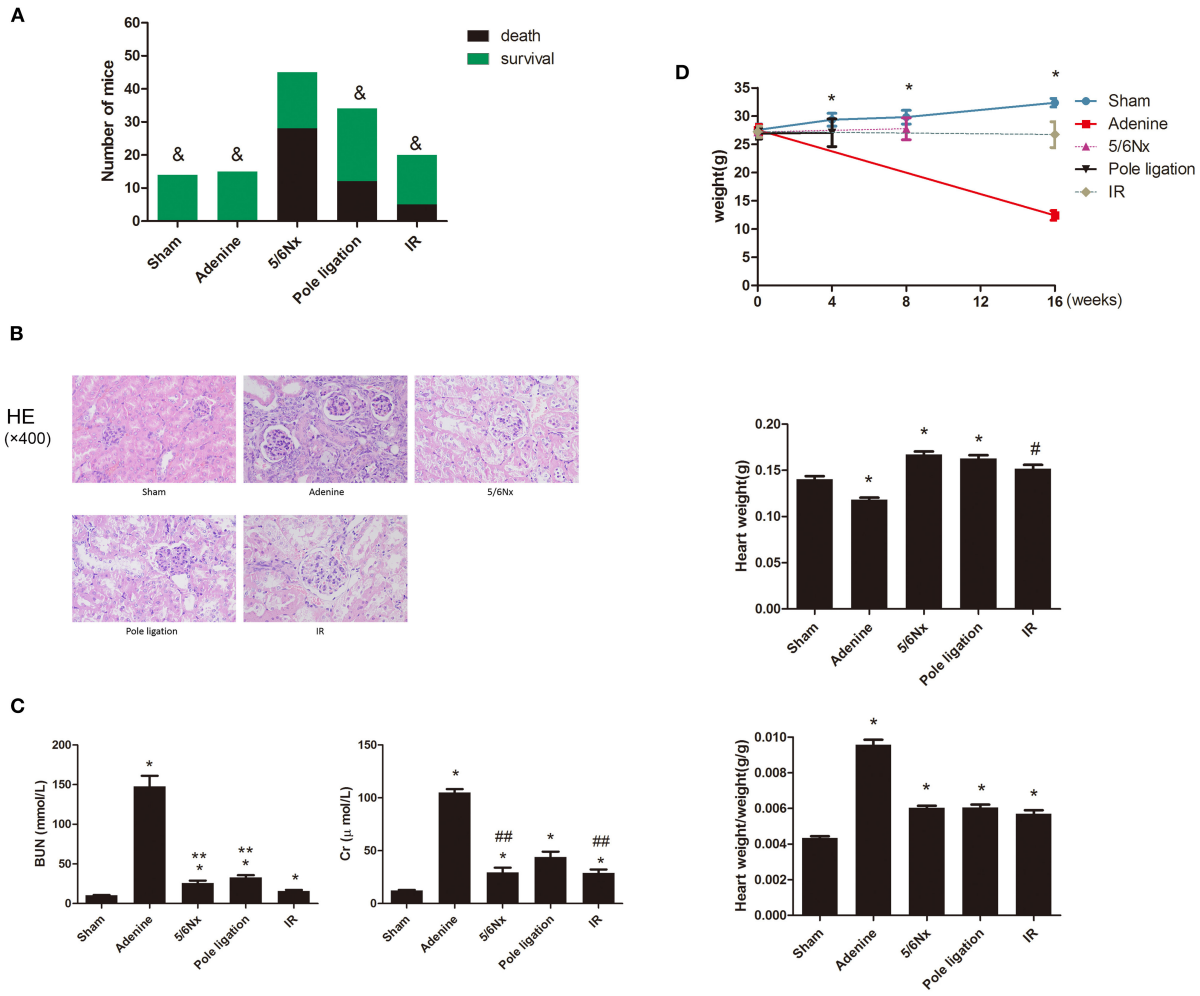
**(A–C)** Between-group comparisons of survival rate, kidney pathology, and renal function. **(A)** Comparisons of survival rates in the sham group, adenine group, 5/6 nephrectomy group (5/6Nx), pole ligation group, and uninephrectomy plus contralateral ischemia followed by reperfusion group (IR). **(B)** Comparisons of renal pathology in different models. **(C)** The mean BUN and Cr in the sham group and modeling groups. Data are presented as the mean ± SEM (*n* = 14–22). ^&^*P* < 0.05, vs. 5/6Nx group; *P < 0.01, vs. Sham; **P < 0.01, vs. IR group; ^#^*P* < 0.05, vs. Sham; ^##^*P* < 0.05, vs. pole ligation group. **(D)** Between-group comparisons of body weight, heart weight, and heart weight-to-body weight ratio. **(D)** The mean body weight, heart weight, and heart weight-to-body weight ratio in the sham, adenine group, 5/6 nephrectomy group (5/6Nx), pole ligation group, and uninephrectomy plus contralateral ischemia followed by reperfusion group (IR). Data are presented as the mean ± SEM (*n* = 14–22). *P < 0.01, vs. Sham; ^#^*P* < 0.05, vs. Sham.

Eight-week-old mice were randomly assigned to the following experimental groups: sham groups (*n* = 14), 5/6 nephrectomy group (5/6Nx) (*n* = 45) (Figure 1A,a), pole ligation group (*n* = 34) (Figure 1A,b), uninephrectomy plus contralateral ischemia followed by reperfusion group (IR) (*n* = 20) (Figure 1A,c), and adenine group (*n* = 15). After modeling, the final study sample comprised 83 mice: 14 in the sham group (survival rate 100%), 15 in the adenine group (survival rate 100%), 17 in the 5/6Nx group (survival rate 37.7%), 22 in the pole ligation group (survival rate 64.7%), and 15 in the IR group (survival rate 75%) (Figure 2A). The mortality in the 5/6 Nx group was significantly higher than that in the pole ligation, IR, and adenine groups. Renal histology indicated glomerular hypertrophy, renal tubular casts, and renal tubular epithelial cell injury in the adenine group, 5/6Nx group, pole ligation group, and IR group (Figure 2B). BUN and Cr were significantly higher in the four modeling groups than in the sham group. In addition, the BUN and Cr levels in the adenine group were also significantly higher than those in the 5/6Nx group, IR group, and ligation group. Interestingly, BUN and Cr levels in the pole ligation group were only slightly higher than those in the 5/6Nx group and IR group (Figure 2C).

### Comparisons of Body Weight (BW) and Heart Weight in Different Models

After surgery or an adenine diet, body weight in the adenine group, 5/6Nx group, pole ligation group, and IR group decreased compared to that in the sham group, and weight loss in the adenine group was particularly significant (Figure 2D). There was a significantly greater heart weight and higher ratio of heart weight to body weight in the 5/6Nx group, pole ligation group, and IR group than in the sham group. Although the heart weight of the adenine group was slightly less than that of the sham group, the heart weight-to-body weight ratio was significantly higher (Figure 2D).

### Cardiac Histology Changes in Different Models

**Figure 3:**
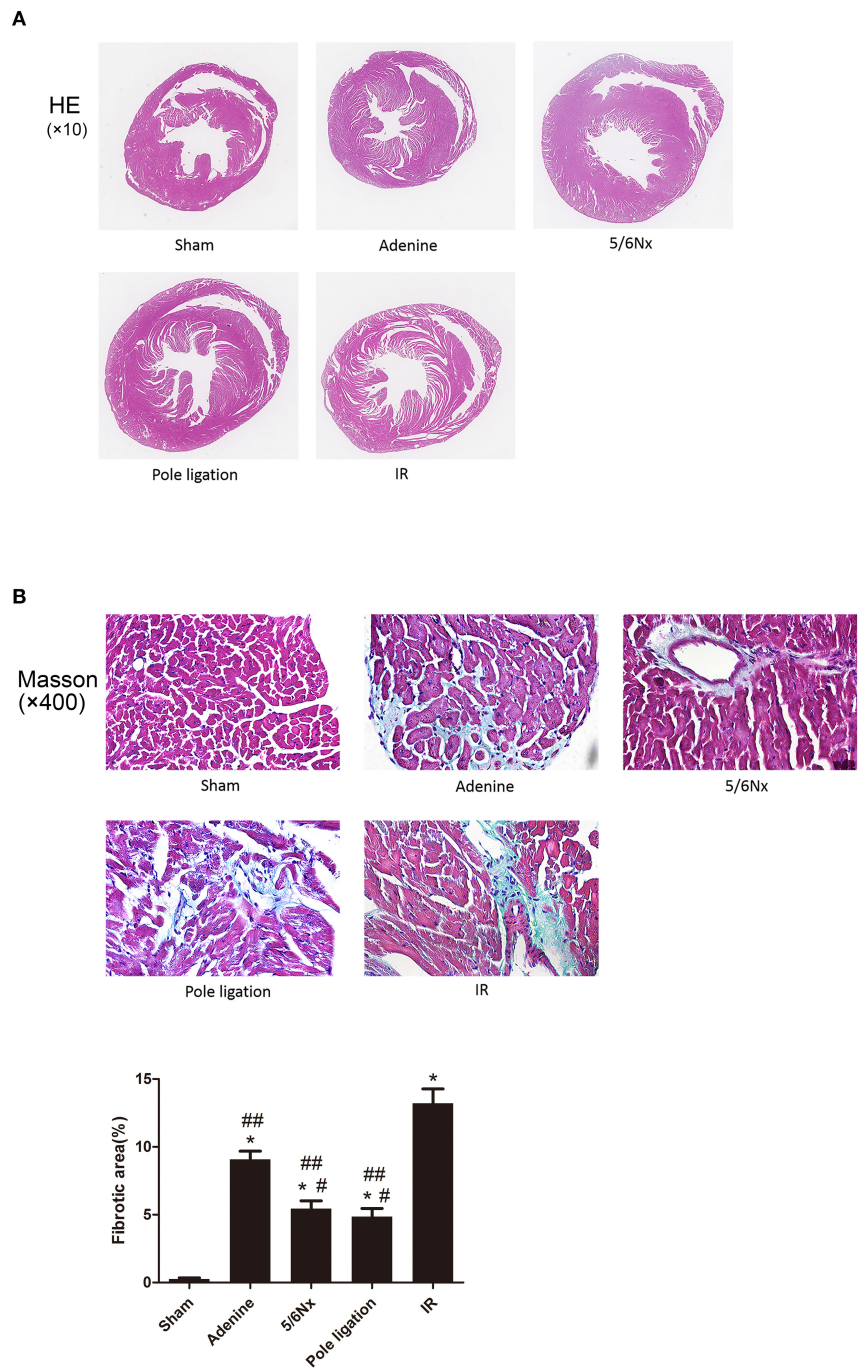
Comparisons of cardiac histology changes in different models in CKD mice. **(A)** Representative micrographs of transverse sections (HE). **(B)** Representative micrographs of left ventricular sections (Masson). *P < 0.001, vs. Sham; ^#^*P* < 0.01 vs. adenine group; ^##^*P* < 0.001 vs. IR group.

A majority of CKD mice developed cardiac hypertrophy. We observed significant increases in ventricular wall thickness in the 5/6Nx groups, pole ligation model group, and IR group mice compared with the sham group via HE staining. Interestingly, the whole heart got bigger in the 5/6Nx group, pole ligation group, and IR group, but slightly reduced in the adenine group. We also observed significant increases in ventricular wall thickness relative to the heart volume in adenine group (Figure 3A). Because cardiac fibrosis plays an important role in the development of UCM, we performed Masson staining on myocardial tissue and found that compared with the sham group, the adenine group, 5/6Nx group, pole ligation group, and IR group all had obvious myocardial interstitial fibrosis. In addition, cardiac fibrosis was more severe in the IR and adenine groups (Figure 3B).

### Comparisons of Cardiac Echocardiography and Doppler in Different Models

**Figure 4:**
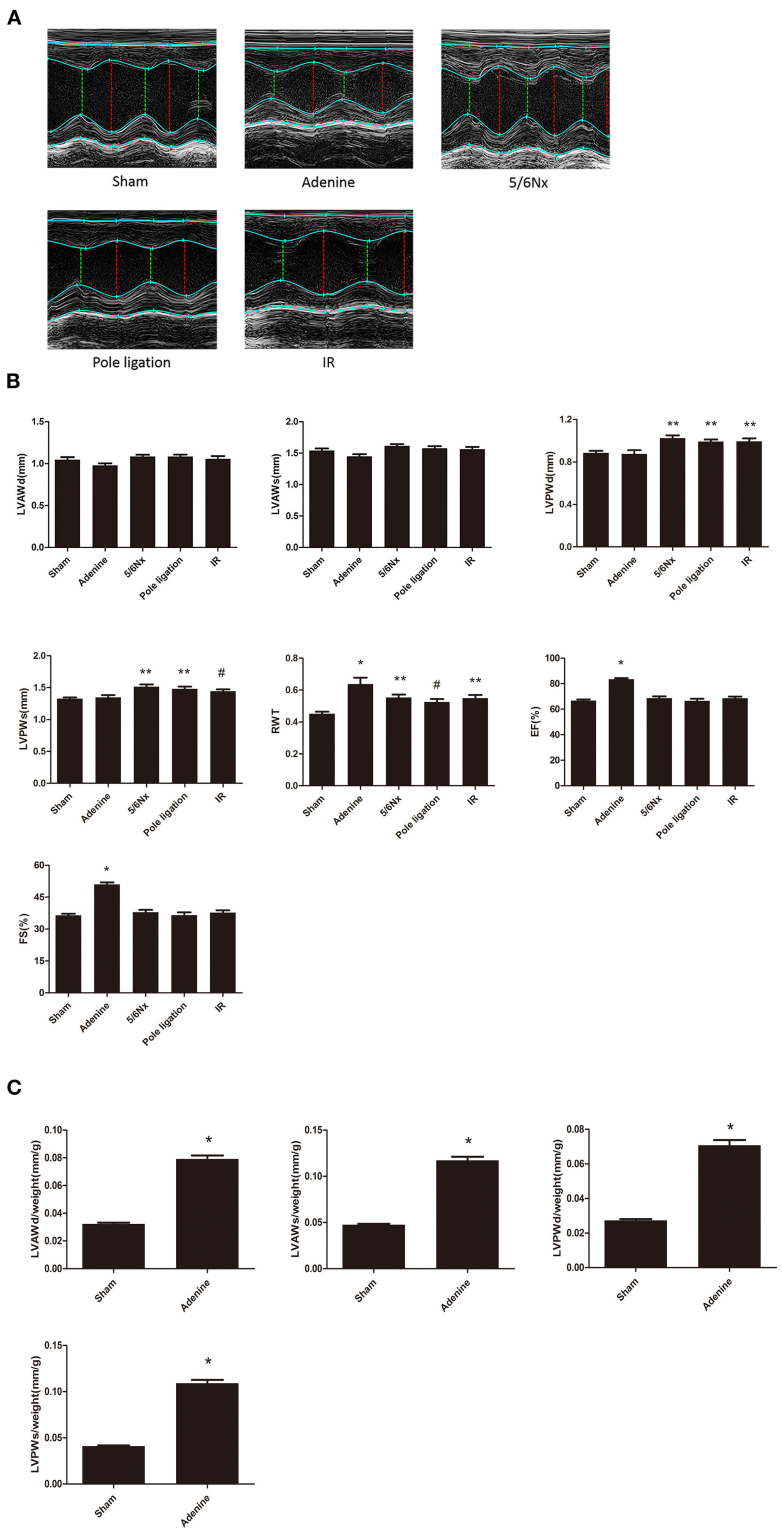
Between-group comparisons of cardiac characteristics in echocardiography. **(A)** Representative M-mode echocardiograms from the sham, adenine group, 5/6th nephrectomy group (5/6Nx), pole ligation group, and uninephrectomy plus contralateral ischemia followed by reperfusion group (IR); **(B)** The mean LVAWS, LVPWs, LVPWs, LVPWd, RWT, LVEF, and LVFS in the five groups. Data are presented as the mean ± SEM (*n* = 14–22). **(C)** The mean LVAWs/BW, LVPWs/BW, LVPWs/BW, and LVPWd/BW in the sham and adenine groups. Data are presented as the mean ± SEM (*n* = 14–15). *P < 0.001, vs. Sham; **P < 0.01, vs. Sham; ^#^*P* < 0.05, vs. Sham.

**Figure 5:**
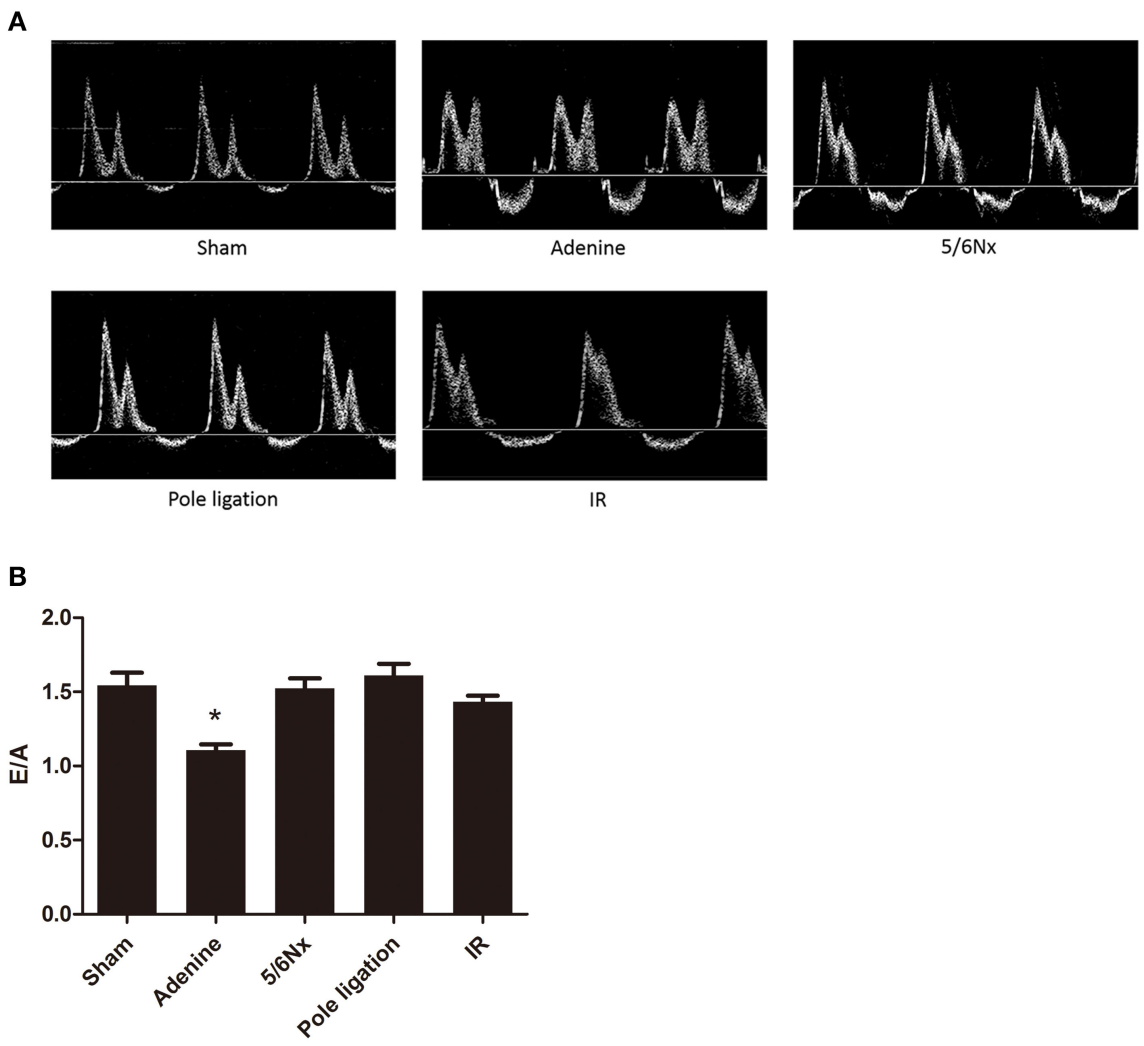
Between-group comparisons of left ventricular diastolic function assessed by echocardiography and Doppler. **(A)** Representative M-mode echocardiograms from the sham, adenine group, 5/6th nephrectomy group (5/6Nx), pole ligation group, and uninephrectomy plus contralateral ischemia followed by reperfusion group (IR). **(B)** The mean E/A in the sham, adenine group, 5/6th nephrectomy group (5/6Nx), pole ligation group, and uninephrectomy plus contralateral ischemia followed by reperfusion group (IR). Data are presented as the mean ± SEM (*n* = 14–22). *P < 0.001, vs. Sham.

Echocardiography showed that LVPWs, LVPWd, and RWT in the 5/6 Nx group, pole ligation group, and IR group were significantly thicker than those in the sham group, suggesting cardiac hypertrophy in the CKD mouse model (Figures 4A,B). The weight of mice in the adenine group was less than half of that in the sham group (sham group weight vs. adenine group weight: 32.378 ± 0.740 g vs. 12.413 ± 0.856 g, *P* = 0.000). We further evaluated the differences in the ratio of LVAWs, LVAWd, LVPWs, and LVPWd to body weight between the adenine group and sham group and found that the above parameters were much higher in the adenine group (Figure 4C). In addition, LVEF and FS in the adenine group were higher than those in the sham group; however, there were no significant differences in these parameters among the 5/6Nx groups, pole ligation group, IR group and sham group (Figure 4B). Analysis of diastolic function revealed no change in the E/A ratio between the sham group and the 5/6Nx group, pole ligation model group, and IR group. However, compared with the sham group, the adenine group showed a significant reduction in the E/A ratio (Figures 5A,B).

### Myocardial FGF-23 and GDF-15 Protein Expression in Heart Tissue

**Figure 6:**
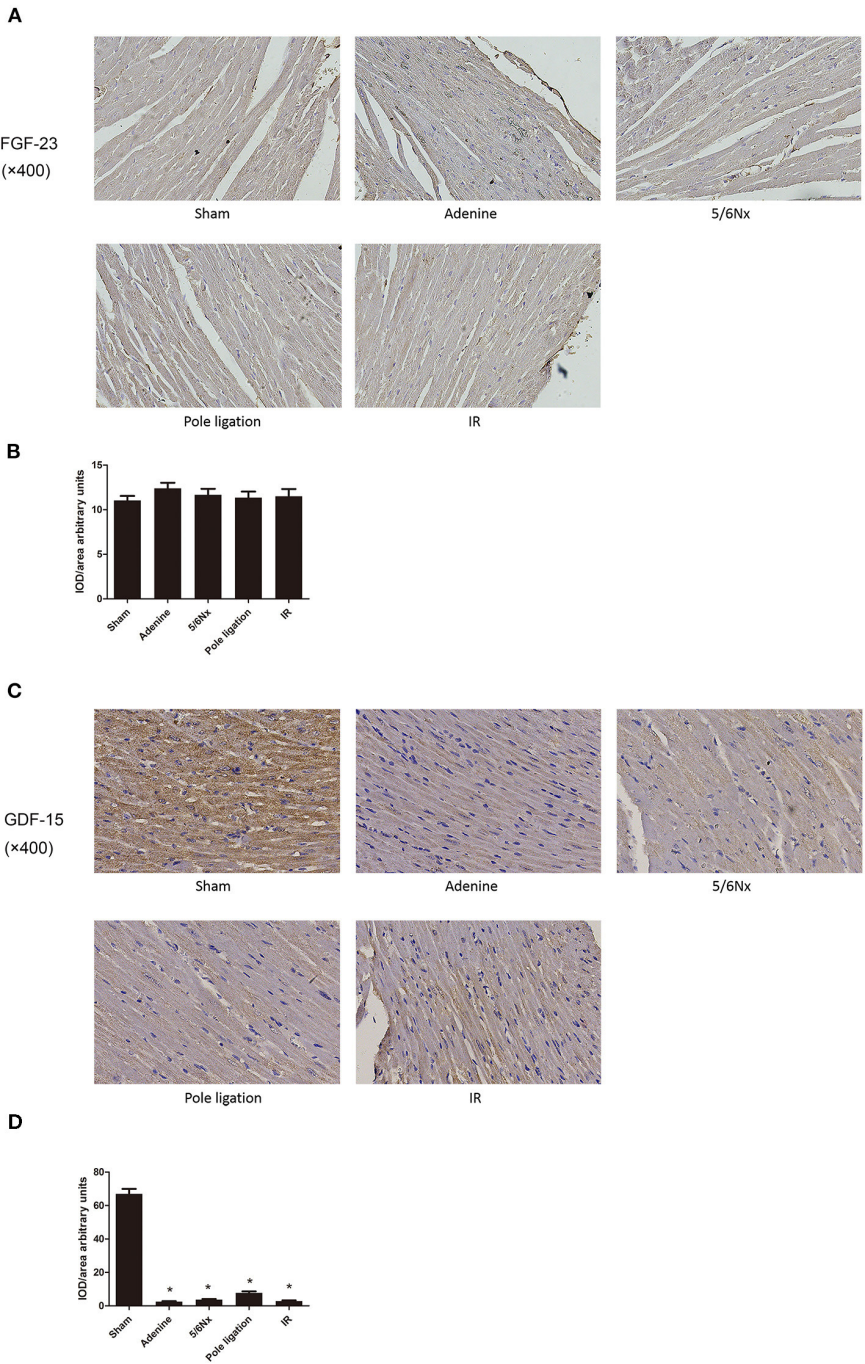
Comparisons of myocardial FGF-23 and GDF-15 protein expression. **(A)** Representative immunohistochemical staining of FGF-23. **(B)** Relative values of FGF-23 optical density according to groups. **(C)** Representative immunohistochemical staining of GDF-15. **(D)** Relative values of GDF-15 optical density according to groups. *P < 0.001, vs. Sham.

Cardiac FGF-23 immunostaining was similar among the five groups (Figures 6A,B). However, immunohistochemistry of the heart tissue showed that the expression of GDF-15 decreased in the 5/6Nx group, adenine group, pole ligation group, and IR group compared to the sham group (Figures 6C,D).

## Discussion

Chronic kidney disease is a significant medical problem globally (14). Indeed, the majority of patients with CKD will die from cardiovascular disease (CVD) before progressing to end-stage renal disease (ESRD) (15). UCM is a widely prevalent cardiovascular disease in patients with CKD or ESRD, accounting for ~50% of deaths due to CKD (11). UCM in patients with CKD or ESRD leads to LVH, left ventricular dilation, left ventricular systolic and diastolic dysfunction, and cardiac fibrosis. Severe UCM can lead to sudden cardiac death even in those without cardiac symptoms.

Although the rat is an extremely useful model for studying the cardiomyopathy of renal failure (4), mouse UCM models were developed due to the wide availability and ease of genetic manipulation in the mouse system. Because of the much smaller size of the kidney, however, classical surgical modeling is very challenging in mouse models with possible high mortality and unexpected bleeding or blood loss. Several UCM mouse models have been reported, but there has been no research comparing the advantages and disadvantages in different models. We used 5/6Nx, IR, pole ligation and an adenine diet to induce UCM and compared the merits and demerits of several models. Although surgery is difficult and the mortality is high, 5/6 nephrectomy is a classic method of inducing the cardiovascular complications of CKD such as UCM. The basic operation involved removing the upper and lower thirds of the left kidney, keeping the complete renal hilus, and removing the right kidney 1 week later. As a classic modeling method for CKD and its cardiovascular complications, 5/6 nephrectomy has been widely used in a large number of experiments. However, the difficulty of surgery and high mortality after surgery still plague researchers. When Kukida et al. studied CKD vascular calcification, they planned to use a 5/6 Nx and high-phosphorus diet to induce CKD vascular calcification. Because of the high mortality of mice, they had to abandon the surgical modeling method and change to an adenine and high-phosphorus diet to induce vascular calcification (16). To successfully complete the surgery, researchers have to spend more time practicing surgical techniques and increasing the number of experimental animals, which not only prolongs the experimental time but also places pressure on scientific research funds. In our experiments, we found that excessive excision of the upper and lower poles of the kidney and a remaining left kidney mass <30% will significantly increase mortality. In addition, the haemostasis effect of the cut surface also has a significant impact on the survival rate of mice. For this reason, we used arterial clips to close the renal artery when removing the upper and lower poles of the kidney and used electric cautery to stop bleeding of the cut surface. After 8 weeks, cardiac echocardiography indicated that LVPWd, LVPWs, and RWT were greater in the surgery group than in the sham group, suggesting that 5/6 Nx mice developed LVH. Heart failure with preserved ejection fraction (HFpEF) characterized by LVH is a common cardiovascular complication of CKD (17). In our study, cardiac echocardiography showed no significant difference in LVEF and FS between the 5/6 Nx group and the sham group. In addition, HE staining showed that the heart was significantly enlarged and that the ventricular wall in 5/6 Nx mice was significantly thicker than that in the sham group, and Masson staining showed that myocardial fibrosis occurred in the 5/6 Nx group.

Recently, Wang et al. reported that the pole ligation method could induce UCM (12). The upper and lower poles of the left kidney were ligated gently with sutures, resulting in ischaemia of the distal kidney, and the right kidney was removed 1 week later. In comparison with the 5/6 Nx models established by pole resection, the pole ligation method is easy, quick, and less instrumentally demanding. More importantly, we found that the mortality of the pole ligation method was significantly lower than that of the 5/6 Nx procedure. Echocardiography indicated that the LVPWd, LVPWs, and RWT were greater in the surgical group than in the sham group, and there was no significant difference in LVEF and FS between the pole ligation group and the sham group. Furthermore, HE staining showed that the heart was significantly enlarged and that the ventricular wall of pole ligation mice was significantly thicker than that in the sham group. Masson staining showed that myocardial fibrosis occurred in the pole ligation model mice.

Hu et al. reported that ischaemia-reperfusion injury on one side plus nephrectomy on the contralateral side (IR model) also induced UCM (11). During the operation, the left renal artery was clamped for 30 min, causing renal ischaemia-reperfusion injury, and the right kidney was immediately removed. After 16 weeks, echocardiography demonstrated that LVPWd, LVPWs, and RWT were greater in the IR group than in the sham group, which suggested that the IR group mice developed LVH. However, there was no significant difference in LVEF and FS between the IR group and sham group. Cardiac tissue HE staining showed that the ventricular wall of the IR group was significantly thicker than that of the sham group, and Masson staining demonstrated that myocardial fibrosis also occurred in the IR group. In addition, cardiac fibrosis was more serious than 5/6Nx and pole ligation groups.

In addition to the surgical method, another model of CKD is a chemical method involving the addition of adenine to the diet. A large number of studies have used 0.20% dietary adenine for 6 to 8 weeks to induce CKD in mice (18–20). However, this is not long enough for cardiovascular effects to develop. Thus, the diet regimen was extended to 16 weeks, causing the development of UCM. After 16 weeks, the body weight of the adenine group was significantly less than that of the sham group, while the ratio of heart weight to body weight was much higher than that of the sham group. HE staining showed that the heart was slightly reduced in the adenine group, but the ventricular wall of the adenine group was significantly thicker than that in the sham group. Masson staining demonstrated that myocardial fibrosis also occurred. Interestingly, it was more serious than 5/6Nx and pole ligation groups, but not as serious as IR group. Echocardiography suggested that the ratios of LVAWs, LVAWd, LVPWs, and LVPWd to body weight were all significantly higher than those in the sham group. In addition, LVEF and FS were significantly higher in the adenine group than in the sham group, which was different from the 5/6 Nx, pole ligation, and IR groups. Given that the hearts were slightly reduced but the thickness of the ventricular wall was increased significantly in the adenine group, the increase in LVEF and FS did not mean that systolic function improved; rather, it indicated that cardiac hypertrophy was serious. Doppler-derived mitral flow velocities revealed a reduction in the E/A ratio in adenine mice, and such an alteration is always accompanied by diastolic relaxation abnormalities (21). Interestingly, compared with that in the sham group, the E/A ratio did not change in the 5/6Nx, pole ligation, and IR groups. Although the adenine model group is more convenient and heart damage is severe, the potential toxicity of adenine itself to the heart cannot be completely ruled out. More importantly, adenine caused significant weight loss in mice, which may cause metabolic and physiological dysfunction of various organs, resulting cardiac injury indirectly.

Although imaging and histological examination are the main diagnostic criteria of UCM, Atrial natriuretic peptide(ANP) and β-myosin heavy chain (β-MHC) are well known as important biomarkers of heart failure and cardiac hypertrophy (22–24). In present study, we confirmed that four models of UCM mice showed cardiac hypertrophy and myocardial fibrosis by echocardiogram and histological examination. However, we did not detect expression of β-MHC and ANP, which is a limitation of this study.

The pathogenesis of UCM is complex, and the underlying mechanism is incompletely understood. It is thought that traditional cardiovascular risk factors, such as hypertension, volume overload, and anemia, are major causes of UCM; however, UCM may also develop independently of these above mentioned risk factors. The exact mechanism is still largely unknown. Growth differentiation factor-15 (GDF-15), also known as macrophage inhibitory cytokine-1 (MIC-1), is a novel member of the transforming growth factor β superfamily (25). It was identified in 1997 as a macrophage-derived inflammatory response cytokine (26). A large number of previous studies have reported that elevated levels of GDF-15 in circulation are closely related to cardiovascular death in CKD patients. Benes et al. reported that serum GDF-15 levels were closely related to poor prognosis in CKD patients with systolic dysfunction and heart failure (27). The mortality of patients with high serum GDF-15 was significantly higher than that of dialysis patients with low serum GDF-15 (28, 29). However, Liu et al. found that overexpression of GDF-15 could inhibit neuronal cell damage induced by oligomycin (25). Recently, Coll et al. reported that circulating elevated GDF-15 played an important role in metformin's benefit in terms of energy balance and weight (30). The exact biological function of GDF-15 is still poorly understood, and it exhibits a complex pattern of beneficial and harmful functions. Whether increased expression of GDF-15 can cause direct damage or may represent a protective response to biological stress is still an open question (31). Previous studies on GDF-15 have mainly focused on its expression in serum, and its expression in heart tissue in CKD has not yet been reported. More importantly, overexpression of GDF-15 in neural cells could protect cells from damage. Therefore, it is interesting and meaningful to explore the expression of GDF15 in hearts of UCM mice. We found that the expression of GDF-15 was significantly higher in the heart tissue of the sham group than in the same tissue of the adenine group, 5/6Nx group, ligation group, and IR group, indicating that GDF-15 expression downregulation in the heart tissue may be one of the mechanisms of UCM. Downregulation of GDF-15 in heart may be induced by traditional and non-traditional risk factors of cardiovascular complications of CKD. Mitochondrial dysfunction plays an important role in UCM (32), and GDF-15 improves mitochondrial function (22). The down-regulation of GDF-15 expression in the heart may be an important mechanism for the UCM.

A large number of studies have consistently confirmed that serum or plasma FGF-23 in CKD animals or patients is significantly increased (33–36). Cross-sectional studies have revealed that higher plasma or serum FGF-23 is associated with LVH in CKD, which can lead to congestive heart failure and subsequent death (37, 38). However, the results of studies on the expression of FGF-23 protein in hearts with heart failure or LVH are relatively few and inconsistent. Leifheit-Nestler et al. reported that cardiac FGF-23 was excessively high in patients with CKD and that enhanced myocardial expression of FGF-23 strongly correlated with LVH (39). However, a study carried out by Andersen et al. reported that FGF-23 protein expression in heart tissue of heart failure patients was similar to that in the control group (40). The increase in circulatory FGF-23 expression in CKD has been widely reported (33–38). However, there are few studies on the expression of FGF-23 in the heart, and the results are also inconsistent (39, 40). As it remains to be clarified whether the myocardium contributes to the release of FGF-23 in UCM, we aimed to assess FGF-23 protein expression in the cardiac of sham and UCM mice. In our present study, we found that FGF-23 protein expression in heart tissue in CKD mice was similar to that in sham mice, suggesting that the high circulatory FGF-23 in CKD does not cause accumulation of FGF-23 in the heart. FGF-23 activates promoting cardiac hypertrophy gene transcription by binding to FGF receptors (FGFRs) on the surface of cardiomyocytes membrane, instead of directly entering the cells (41). Therefore, the function and activity of the FGFRs may be more important. Considering that the pathogenesis of uremic cardiomyopathy is very complicated, the proteomics study of myocardial tissue may be very helpful for discovering new key pathogenic molecules of UCM.

## Conclusion

The 5/6Nx model, pole ligation model, IR model, and adenine model can induce uraemic cardiomyopathy in mice. The mortality in the 5/6 Nx group is significantly higher than that in other three modeling groups. The ventricular wall thickness in the four modeling groups mice are significant increased. However, the heart size gets bigger in the 5/6Nx group, pole ligation group, and IR group, but slightly reduces in the adenine group. Four modeling groups all have obvious myocardial interstitial fibrosis. In addition, it is more severe in the IR and adenine groups. Echocardiography indicates that the mice in adenine group shows a significant decrease in cardiac diastolic function (Table 1).

**Table 1:** Comparison of characteristics between different models.

**Model**	**The degree of kidney damage**	**Relative ventricular hypertrophy**	**Cardiac diastolic dysfunction**	**Heart size**	**Cardiac fibrosis**	**Mortality**	**Induction Time**	**Difficulty of operation**
5/6Nx	++	+	-	Enlarged	+	++	++	+++
Pole ligation	+++	+	-	Enlarged	+	+	+	++
IR	+	+	-	Enlarged	+++	+	+++	+
Adenine	+++++	++	+	Reduced	++	-	+++	-

In conclusion, researchers can choose the appropriate model according to the modeling time, surgical technique, characteristics of the heart damage, etc. In addition, we also found that the expression of GDF-15 in heart tissue was significantly downregulated in UCM mice compared with sham mice, suggesting that GDF-15 may be a novel molecular target worthy of further study.

## Data Availability Statement

The original contributions presented in the study are included in the article/supplementary material, further inquiries can be directed to the corresponding authors.

## Ethics Statement

The animal study was reviewed and approved by Animals Experiment Ethics Committee of Nanjing Medical University.

## Author Contributions

CC, CXie, HW, LW, and JZ performed the experiments and analyzed the data. HM and CXin edited and revised the manuscript. All authors approved the final version of the manuscript.

## Conflict of Interest

The authors declare that the research was conducted in the absence of any commercial or financial relationships that could be construed as a potential conflict of interest.

## References

[1] Coresh J Selvin E Stevens LA Manzi J Kusek JW Eggers P. Prevalence of chronic kidney disease in the United States. Jama. (2007) 298:2038–47. 10.1001/jama.298.17.2038 17986697

[2] Weiner DE. Public health consequences of chronic kidney disease. Clin Pharmacol Ther. (2009) 86:566–9. 10.1038/clpt.2009.137 19641489 PMC2788514

[3] Collins AJ Foley RN Chavers B Gilbertson D Herzog C Johansen K. ‘United States renal data system 2011 annual data report: atlas of chronic kidney disease & end-stage renal disease in the United States. Am J Kidney Dis. (2012) 59:e1–420. 10.1053/j.ajkd.2011.11.015 22177944

[4] Trespalacios FC Taylor AJ Agodoa LY Bakris GL Abbott KC. Heart failure as a cause for hospitalization in chronic dialysis patients. Am J Kidney Dis. (2003) 41:1267–77. 10.1016/S0272-6386(03)00359-7 12776280

[5] Kennedy DJ Elkareh J Shidyak A Shapiro AP Smaili S Mutgi K. Partial nephrectomy as a model for uremic cardiomyopathy in the mouse. Am J Physiol Renal Physiol. (2008) 294:F450–4. 10.1152/ajprenal.00472.2007 18032546 PMC2742580

[6] Kennedy D Omran E Periyasamy SM Nadoor J Priyadarshi A Willey JC. Effect of chronic renal failure on cardiac contractile function, calcium cycling, and gene expression of proteins important for calcium homeostasis in the rat. J Am Soc Nephrol. (2003) 14:90–7. 10.1097/01.ASN.0000037403.95126.03 12506141

[7] Kennedy DJ Vetteth S Periyasamy SM Kanj M Fedorova L Khouri S. Central role for the cardiotonic steroid marinobufagenin in the pathogenesis of experimental uremic cardiomyopathy. Hypertension. (2006) 47:488–95. 10.1161/01.HYP.0000202594.82271.92 16446397

[8] Li Y Wu J He Q Shou Z Zhang P Pen W. Angiotensin (1-7) prevent heart dysfunction and left ventricular remodeling caused by renal dysfunction in 5/6 nephrectomy mice. Hypertens Res. (2009) 32:369–74. 10.1038/hr.2009.25 19325560

[9] Kuma A Wang XH Klein JD Tan L Naqvi N Rianto F. Inhibition of urea transporter ameliorates uremic cardiomyopathy in chronic kidney disease. FASEB J. (2020) 34:8296–309. 10.1096/fj.202000214RR 32367640 PMC7302978

[10] Wang B Wang ZM Ji JL Gan W Zhang A Shi HJ. Macrophage-derived exosomal mir-155 regulating cardiomyocyte pyroptosis and hypertrophy in uremic Cardiomyopathy. JACC Basic Transl Sci. (2020) 5:148–66. 10.1016/j.jacbts.2019.10.011 32140622 PMC7046511

[11] Hu MC Shi M Gillings N Flores B Takahashi M Kuro OM. Recombinant α-Klotho may be prophylactic and therapeutic for acute to chronic kidney disease progression and uremic cardiomyopathy. Kidney Int. (2017) 91:1104–14. 10.1016/j.kint.2016.10.034 28131398 PMC5592833

[12] Wang X Chaudhry MA Nie Y Xie Z Shapiro JI Liu J. A mouse 5/6th nephrectomy model that induces experimental uremic cardiomyopathy. J Visualized Exp. (2017) 129:55825. 10.3791/55825 29155790 PMC5755318

[13] Kieswich JE Chen J Alliouachene S Caton PW McCafferty K Thiemermann C. A novel model of reno-cardiac syndrome in the c57BL/ 6 mouse strain. BMC Nephrol. (2018) 19:346. 10.1186/s12882-018-1155-3 30509210 PMC6278034

[14] Hallan SI Coresh J Astor BC Asberg A Powe NR Romundstad S. International comparison of the relationship of chronic kidney disease prevalence and ESRD risk. J Am Soc Nephrol. (2006) 17:2275–84. 10.1681/ASN.2005121273 16790511

[15] Berl T Henrich W. Kidney-heart interactions: epidemiology, pathogenesis, and treatment. Clin J Am Soc Nephrol. (2006) 1:8–18. 10.2215/CJN.00730805 17699186

[16] Kukida M Mogi M Kan-No H Tsukuda K Bai HY Shan BS. AT2 receptor stimulation inhibits phosphate-induced vascular calcification. Kidney Int. (2019) 95:138–48. 10.1016/j.kint.2018.07.028 30442332

[17] Sárközy M Gáspár R Zvara Á Siska A Kovári B Szucs G. Chronic kidney disease induces left ventricular overexpression of the pro-hypertrophic microRNA-212. Sci Rep. (2019) 9:1302. 10.1038/s41598-018-37690-5 30718600 PMC6362219

[18] Santana AC Degaspari S Catanozi S Dellê H de Sá Lima L Silva C. Thalidomide suppresses inflammation in adenine-induced CKD with uraemia in mice. Nephrol Dial Transplant. (2013) 28:1140–149. 10.1093/ndt/gfs569 23345625

[19] Lin W Zhang Q Liu L Yin S Liu Z Cao W. Klotho restoration via acetylation of peroxisome proliferation-activated receptor γ reduces the progression of chronic kidney disease. Kidney Int. (2017) 92:669–79. 10.1016/j.kint.2017.02.023 28416226

[20] Zhang Q Liu L Lin W Yin S Duan A Liu Z. Rhein reverses klotho repression via promoter demethylation and protects against kidney and bone injuries in mice with chronic kidney disease. Kidney Int. (2017) 91:144–56. 10.1016/j.kint.2016.07.040 27692562

[21] Oh JK Appleton CP Hatle LK Nishimura RA Seward JB Tajik AJ. The noninvasive assessment of left ventricular diastolic function with two-dimensional and doppler echocardiography. J Am Soc Echocardiogr. (1997) 10:246–70. 10.1016/S0894-7317(97)70062-2 9109691

[22] Chua S Lee FY Chiang HJ Chen KH Lu HI Chen YT. The cardioprotective effect of melatonin and exendin-4 treatment in a rat model of cardiorenal syndrome. J Pineal Res. (2016) 61:438–56. 10.1111/jpi.12357 27465663

[23] Ogawa N Komura H Kuwasako K Kitamura K Kato J. Plasma levels of natriuretic peptides and development of chronic kidney disease. BMC Nephrol. (2015) 16:171. 10.1186/s12882-015-0163-9 26499263 PMC4620018

[24] Yang K Wang C Nie L Zhao X Gu J Guan X. Klotho protects against indoxyl sulphate-induced myocardial hypertrophy. J Am Soc Nephrol. (2015) 26:2434–46. 10.1681/ASN.2014060543 25804281 PMC4587686

[25] Liu H Liu J Si L Guo C Liu W Liu Y. GDF-15 promotes mitochondrial function and proliferation in neuronal HT22 cells. J Cell Biochem. (2019) 120:10530–47. 10.1002/jcb.28339 30635935

[26] Bootcov MR Bauskin AR Valenzuela SM Moore AG Bansal M He XY. MIC-1, a novel macrophage inhibitory cytokine, is a divergent member of the TGF-beta superfamily. Proc Natl Acad Sci USA. (1997) 94:11514–9. 10.1073/pnas.94.21.11514 9326641 PMC23523

[27] Benes J Kotrc M Wohlfahrt P Conrad MJ Franekova J Jabor A. The role of gDF-15 in heart failure patients with chronic kidney disease. Can J Cardiol. (2019) 35:462–70. 10.1016/j.cjca.2018.12.027 30935637

[28] Breit SN Carrero JJ Tsai VW Yagoutifam N Luo W Kuffner T. Macrophage inhibitory cytokine-1 (MIC-1/GDF15) and mortality in end-stage renal disease. Nephrol Dial Transplant. (2012) 27:70–5. 10.1093/ndt/gfr575 21940482

[29] Tuegel C Katz R Alam M Bhat Z Bellovich K de Boer I. GDF-15, galectin 3, soluble ST2, and risk of mortality and cardiovascular events in CKD. Am J Kidney Dis. (2018) 72:519–28. 10.1053/j.ajkd.2018.03.025 29866459 PMC6153047

[30] Coll AP Chen M Taskar P Rimmington D Patel S Tadross JA. GDF15 mediates the effects of metformin on body weight and energy balance. Nature. (2020) 578:444–8. 10.1038/s41586-019-1911-y 31875646 PMC7234839

[31] Kramer F Milting H. Novel biomarkers in human terminal heart failure and under mechanical circulatory support. Biomarkers. (2011) 16:S31–41. 10.3109/1354750X.2011.561498 21707442

[32] Taylor D Bhandari S Seymour AL. Mitochondrial dysfunction in uremic cardiomyopathy. Am J Physiol Renal Physiol. (2015) 308:579–87. 10.1152/ajprenal.00442.2014 PMC4360036 25587120

[33] Isakova T Wahl P Vargas GS Gutiérrez OM Scialla J Xie H. Fibroblast growth factor 23 is elevated before parathyroid hormone and phosphate in chronic kidney disease. Kidney Int. (2011) 79:1370–8. 10.1038/ki.2011.47 21389978 PMC3134393

[34] Portale AA Wolf M Jüppner H Messinger S Kumar J Wesseling-Perry K. Disordered FGF23 and mineral metabolism in children with CKD. Clin J Am Soc Nephrol. (2014) 9:344–53. 10.2215/CJN.05840513 24311704 PMC3913243

[35] Mulay SR Eberhard JN Pfann V Marschner JA Darisipudi MN Daniel C. Oxalate-induced chronic kidney disease with its uremic and cardiovascular complications in C57BL/6 mice. Am J Physiol Renal Physiol. (2016) 310:F785–95. 10.1152/ajprenal.00488.2015 26764204 PMC5504458

[36] Sugiura H Matsushita A Futaya M Teraoka A Akiyama KI Usui N. Fibroblast growth factor 23 is upregulated in the kidney in a chronic kidney disease rat model. PLoS ONE. (2018) 13:e0191706. 10.1371/journal.pone.0191706 29518087 PMC5843171

[37] Gutiérrez OM Mannstadt M Isakova T Rauh-Hain JA Tamez H Shah A. Fibroblast growth factor 23 and mortality among patients undergoing hemodialysis. N Engl J Med. (2008) 359:584–92. 10.1056/NEJMoa0706130 18687639 PMC2890264

[38] Gutiérrez OM Januzzi JL Isakova T Laliberte K Smith K Collerone G. Fibroblast growth factor 23 and left ventricular hypertrophy in chronic kidney disease. Circulation. (2009) 119:2545–52. 10.1161/CIRCULATIONAHA.108.844506 19414634 PMC2740903

[39] Leifheit-Nestler M Große Siemer R Flasbart K Richter B Kirchhoff F Ziegler WH. Induction of cardiac FGF23/FGFR4 expression is associated with left ventricular hypertrophy in patients with chronic kidney disease. Nephrol Dial Transplant. (2016) 31:1088–99. 10.1093/ndt/gfv421 26681731 PMC6388939

[40] Andersen IA Huntley BK Sandberg SS Heublein DM Burnett JC Jr. Elevation of circulating but not myocardial FGF23 in human acute decompensated heart failure. Nephrol Dial Transplant. (2016) 31:767–72. 10.1093/ndt/gfv398 26666498

[41] Jonathan P Anna M Luke Ashwin R Chris W Jones AM. Clinical potential of targeting fibroblast growth factor-23 and αKlotho in the treatment of uremic cardiomyopathy. J Am Heart Assoc. (2020) 9:e016041. 10.1161/JAHA.119.014566 32212912 PMC7428638

